# A novel homozygous variant of *COL2A1* in a Chinese male with type II collagenopathy: a case report

**DOI:** 10.1186/s12920-021-01048-0

**Published:** 2021-08-11

**Authors:** Qianwen Zhang, Ruen Yao, Qun Li, Xin Li, Biyun Feng, Guoying Chang, Jian Wang, Xiumin Wang

**Affiliations:** 1 Department of Endocrinology, Genetics and Metabolism, Shanghai Children’s Medical Center, School of Medicine, Shanghai Jiao Tong University, Shanghai, China; 2 Department of Medical Genetics and Molecular Diagnostic Laboratory, Shanghai Children’s Medical Center, School of Medicine, Shanghai Jiao Tong University, Shanghai, China; 3 Center for Brain Science Shanghai Children’s Medical Center, School of Medicine, Shanghai Jiao Tong University, Shanghai, China

**Keywords:** Type II collagenopathies, *COL2A1* gene, Whole-exome sequencing, Novel variant, Rare complex syndrome

## Abstract

**Background:**

Type II collagenopathies are a spectrum of diseases and skeletal dysplasia is one of the prominent features of collagenopathies. Molecular defects of the *COL2A1* gene cause type II collagenopathies that is mainly an autosomal dominant disease, whereas some rare cases with autosomal recessive inheritance of mode have also been identified.

**Case presentation:**

The patient was a 5-year-old male with a short neck, flat face, epiphyseal dysplasia, irregular vertebral endplates, and osteochondritis. Sequencing result indicated NM_001844.4: c.3662C > T; p. (Ser1221Phe) a novel missense variant, leading to a serine-to-phenylalanine substitution. Sanger sequencing confirmed the variant compared to his parents and brother.

**Conclusions:**

We identified a novel homozygous variant of the *COL2A1* gene as the cause of type II collagenopathies in a Chinese male, enriching the spectrum of genotypes. This is the first case of type II collagenopathies inherited in an autosomal recessive manner in China and East Asia, and it is the first case that resulted from serine substitution in the world.

**Supplementary Information:**

The online version contains supplementary material available at 10.1186/s12920-021-01048-0.

## Background

Type II collagenopathies are a series of diseases characterized by skeletal dysplasia [1]. The clinical symptoms are notably variable, including short stature, eyesight abnormality, hearing loss, kyphosis, and epiphyseal dysplasia. According to the variety and severity of their symptoms, patients are classified into 21 different phenotypes [2] such as Stickler syndrome type I (STL1, MIM#108300) and Spondyloepiphyseal dysplasia congenita (SEDC, MIM#183900), though clinical presentations of these phenotypes overlap considerably. Type II collagenopathies are resulted by mutation of *COL2A1* (MIM #120140) and are inherited in an autosomal dominant manner. Recently, autosomal recessive inherence was identified in patients with type II collagenopathies: all of the patients were from West Asia [3–6], probably owing to a higher rate of consanguinity and extensive development of gene sequencing.

The *COL2A1* gene, which is located in the 12q13.11 region, contains 54 exons and encodes the alpha-1 chain of procollagen type II [7]. It is specifically expressed in the vitreous, cartilage, inner ear, and intervertebral discs, which explains the typical clinical features of type II collagenopathies [8, 9]. The protein encoded by *COL2A1* contains a typical triple-helical domain and a C-terminal region where variants have been found previously. The triple-helical domain comprises Gly-X-Y repeats, which is a typical feature of collagens. Most variants found in patients with type II collagenopathies are located in the triple-helical domain and Gly substitutions in this domain are usually related to severe phenotypes [2].

In the present study, whole-exome sequencing (WES) was performed on DNA sample obtained from one Chinese patient manifesting a short neck, flat face, epiphyseal dysplasia, irregular vertebral endplates, and osteochondritis. A novel homozygous variant of the *COL2A1* gene was identified and the patient was diagnosed with type II collagenopathy. This is the first case of a patient with a *COL2A1* homozygous variant in China and in East Asia. It is also the first case that resulted from a serine substitution worldwide.

## Case presentation

The patient was a 5-year-old male with a birth length of 51 cm (+ 0.33SD) and a birth weight of 4000 g (+ 1.6SD). He is the second child of a non-consanguineous healthy couple and was delivered by caesarean section at 40 weeks. Development was normal before one and a half years of age; subsequently, lower-limb abnormality and short stature were noticed. Since three years of age, he has been suffered from consistent low bone mineral density (Z:1.6 ~ − 2.4, 1–5%) which could not be corrected by calcium and vitamin D supplementation. He occasionally complained of ankle ache after exercise. At 5 years of age, he was referred to our department due to the manifested bone abnormality.

**Fig. 1:**
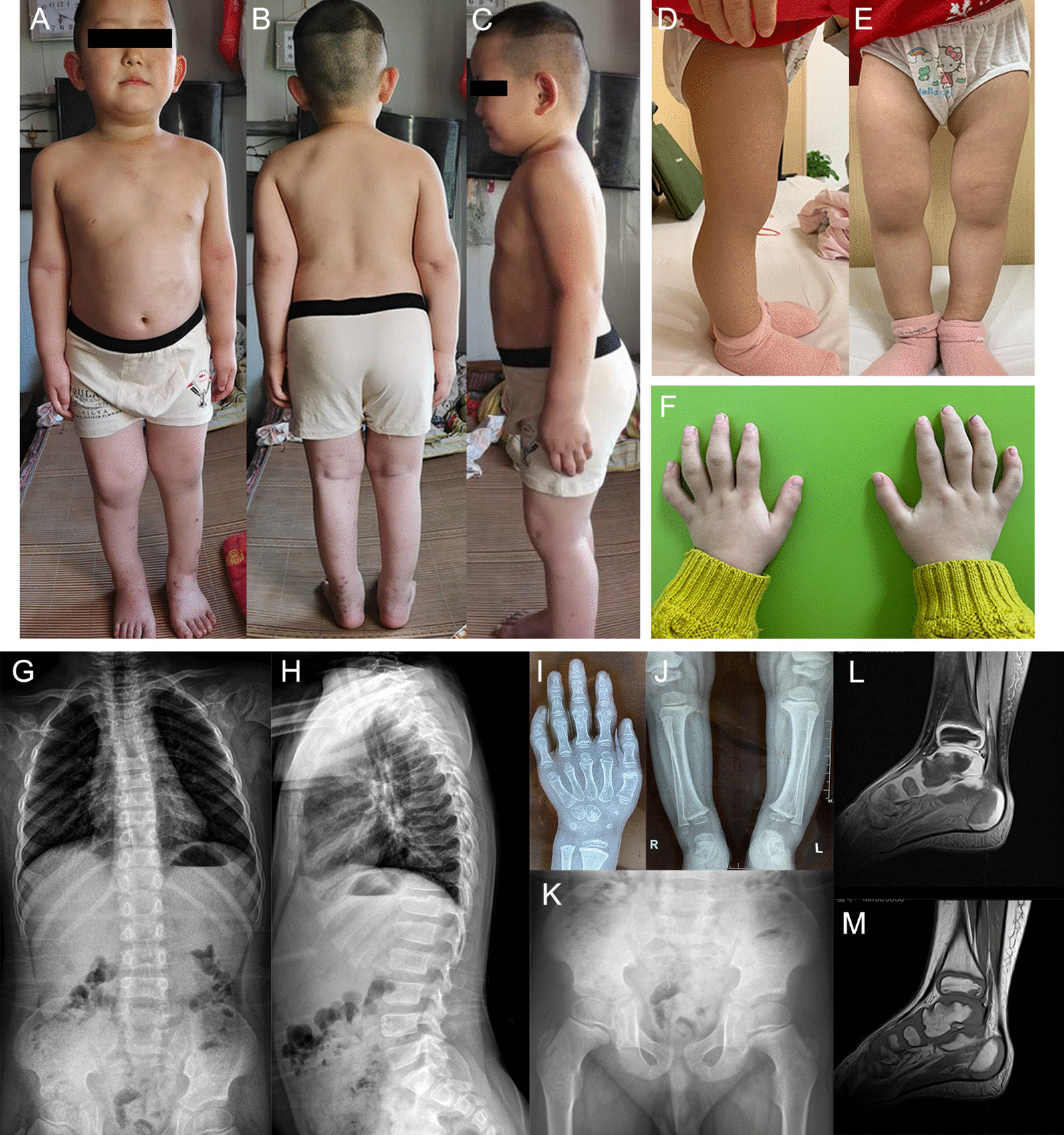
Clinical photographs of the patient. **A**–**C** Facial characteristics including flat face and short neck. **D**, **E** Short lower limbs. **F** Brachydactyly and enlargement of medium interphalangeal joints. **G**, **H** Platyspondyly with irregular vertebral endplates. **I**, **J** Metaphyseal enlargement of medium interphalangeal and knee joints. **L** Heterogeneous bone structure of the proximal femur. **K**, **M** Osteochondritis of the right ankle

Physical examination showed a flat face, short neck, short lower limbs especially under the knees, brachydactyly, enlargement of the medium interphalangeal joints with normal height (105.8 cm, − 1.29SD) and weight (24 kg, + 1.87SD) (Fig. 1A–F). The patient had normal hearing, vision, cognitive development, and expression ability. Chest computed tomography and urinary system ultrasound were normal. X-ray of the spine showed that platyspondyly with irregular vertebral endplates of the lumbar vertebra and thoracic vertebra, and bony defects were observed on the top of T12 (Fig. 1G, H). X-ray of the knees and hand confirmed the results found on physical examination (Fig. 1I, J) and that of the pelvis revealed a heterogeneous bone structure of the proximal femur (Fig. 1K). Magnetic resonance imaging of the right ankle showed signs of osteochondritis (Fig. 1L, M).

Laboratory investigations revealed normal levels of liver function, renal function, erythrocyte sedimentation rate, thyroid function, and levels of parathyroid hormone and serum calcium. Additionally, the patient showed elevated levels of alkaline phosphatase (184 IU/L, reference range: 45–129 IU/L) and serum inorganic phosphorus (1.8 mmol/L, reference range: 0.78–1.65 mmol/L). Further, myocardium zymogram examination revealed that creatine kinase isoenzyme-MB (4.8 μg/L, reference range: < 3.7 μg/L) and creatine kinase isoenzyme (193 U/L, reference range: 55–170 U/L) levels were above the normal.

**Fig. 2:**
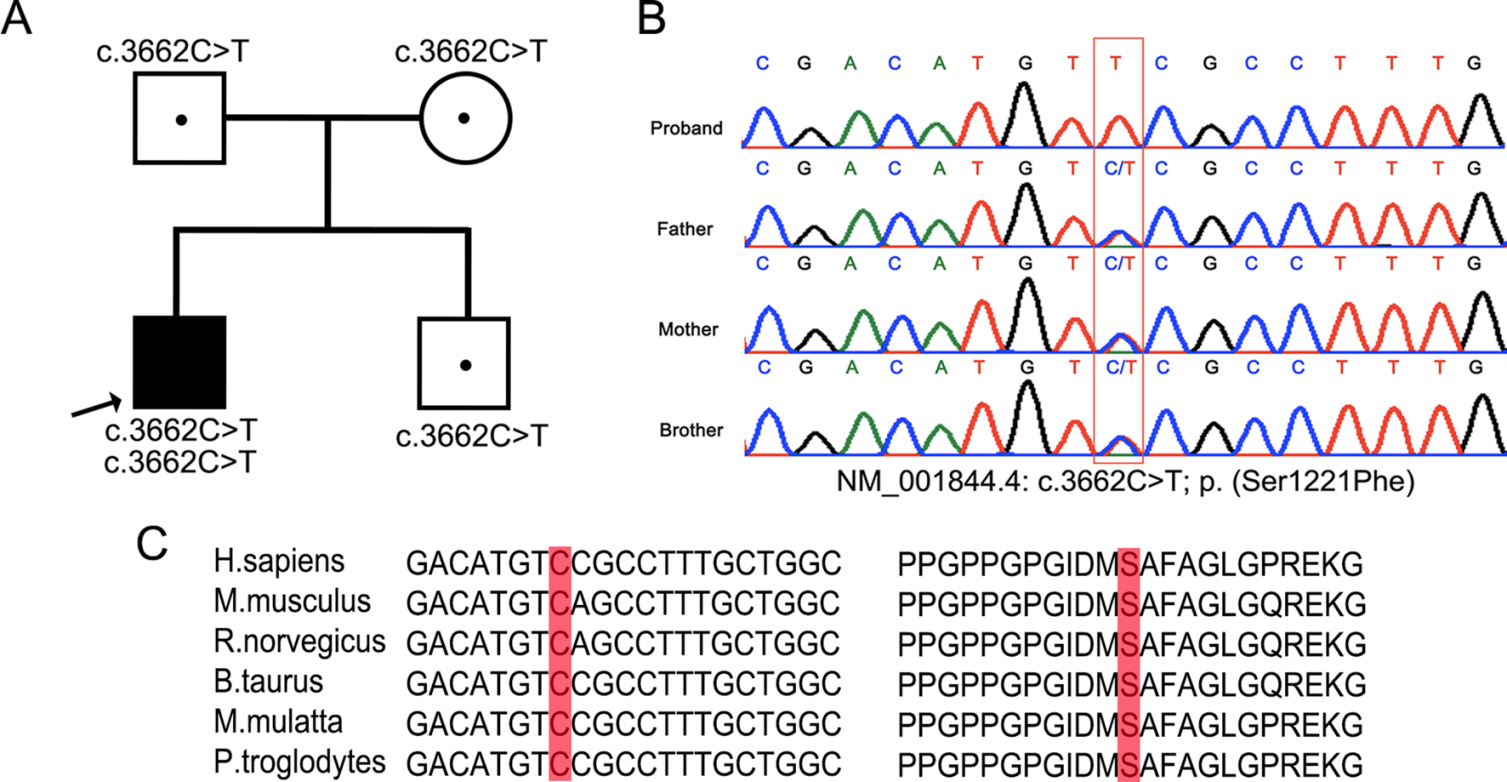
Verification and functional prediction of the c.3662C > T in the *COL2A1* gene. **A** Pedigree of the family. **B** Sanger sequencing showed a homozygous missense variant in the patient, and the proband’s parents and brother were all heterozygous of the same locus. Black arrows, mutant base. **C** The position of the mutant residue, indicated in red, was highly conserved

The patient’s father was 173 cm, and his mother was 156 cm in height; both parents were healthy. He also had an 8-year-old healthy brother, with a height of 130.5 cm (Fig. 2A). The parents’ and the only brother's spines, pelvises, and knees were evaluated using X-ray and no abnormality was observed (Additional file 1: Figs. S1, S2, S3).

WES was performed on the proband. Sequencing was performed and clusters were generated with an Illumina HiSeq 2000 system (Illumina, Inc.) and an Illumina cBot system (Illumina Inc., San Diego, CA, USA) respectively. The average read depth was 162.91X (Additional file 1: Table S1). All variants were annotated and filtered by Ingenuity Variant Analysis (Ingenuity Systems, Redwood City, CA, USA). Candidate variants were analyzed while skeletal dysplasia was selected as the main filtering symptom. Among all filtered variants, a homozygous missense variant in *COL2A1* was confirmed and explained the patient’s condition (PM2 + PP3 + PP4), according to the guidelines recommended by the American College of Medical Genetics and Genomics (ACMG) (Additional file 1: Table S2). The novel variant NM_001844.4: c.3662C > T in *COL2A1* led to a serine-to-phenylalanine substitution. Sanger sequencing indicated that the parents and brother were heterozygous for this variant (Fig. 2A, B).

**Fig. 3:**
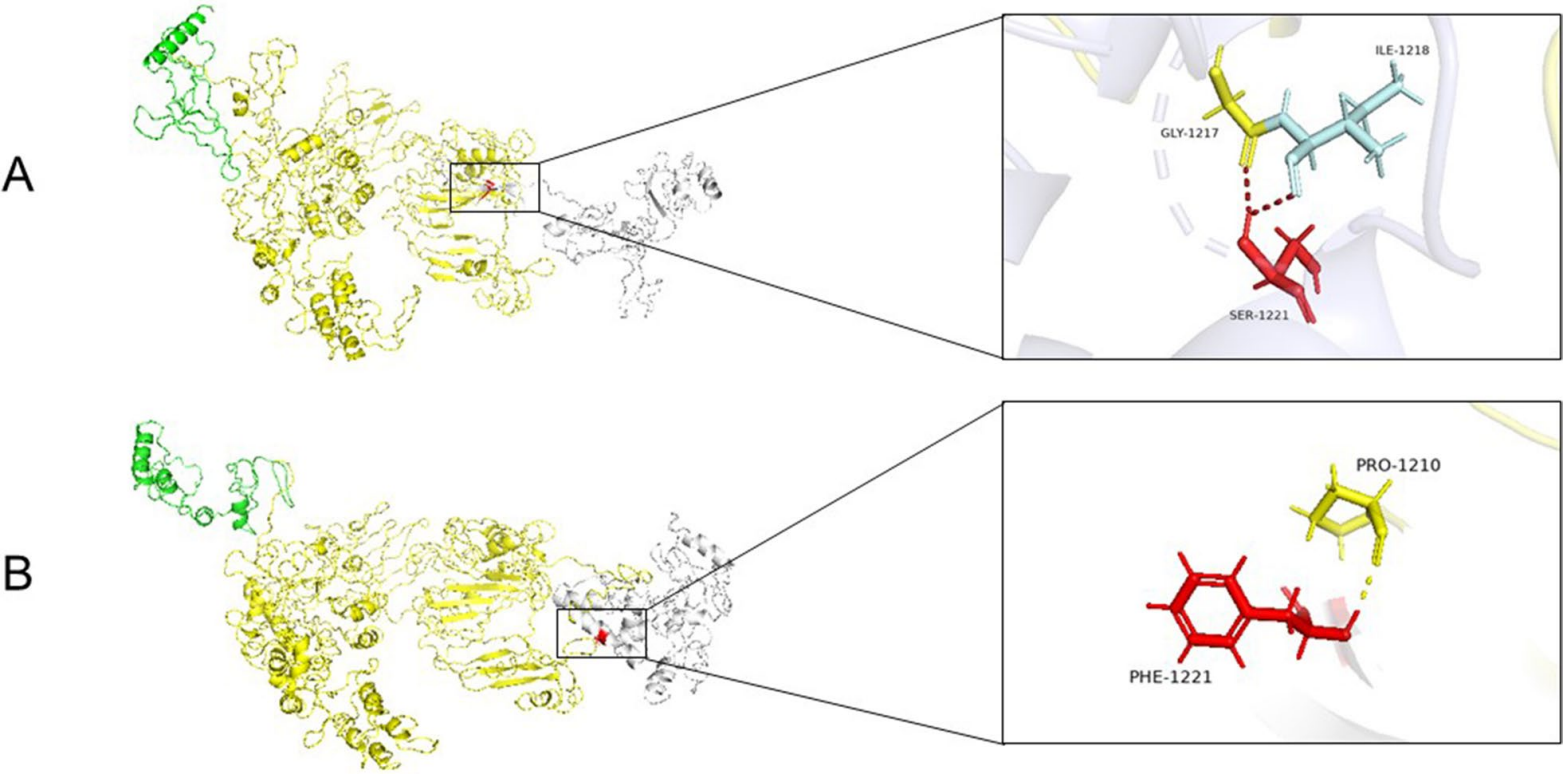
Three-dimensional structure model of the WT COL2A1 and p. (Ser1221Phe) mutant. The Triple helical domain and C-terminal propeptide are shown in yellow and white respectively. **A** Wild type protein: Serine at 1221 (red) interacts with Glycine at 1217 and Isoleucine at 1218. **B** Mutant protein: Serine at 1221 (red) interacts only with Proline at 1210

Using in silico tools, we evaluated the pathogenicity of the variant of the *COL2A1.* The position of the variant is highly conserved in multiple species (Fig. 2C). Functional prediction indicated that the variant has a deleterious effect on the protein according to PolyPhen-2 (probably damaging, score = 0.99), SIFT (damaging, score = 0.003), and MutationTaster (disease causing, score = 1). To better assess the pathogenicity, a three-dimensional model was generated and examined using the I-TASSER server[10] (http://zhanglab.ccmb.med.umich.edu/I-TASSER) and Pymol v.1.8.4.0 software (https://www.pymol.org; Schrödinger, New York, NY, USA) respectively (Fig. 3). Normally, the amino acid residue serine is located in the C-terminal propeptide, which participates in the formation of an α-helix. The variant c.3662C > T is indicated to alter the hydrogen bond resulting in disruption of the normal protein structure. The patient was finally diagnosed with type II collagenopathy caused by a novel homozygous variant in *COL2A1*.

## Discussion and conclusions

In the present study, since the observed phenotype was not completely typical for collagenopathy, WES was considered for diagnosis based on its wide coverage and not cost-incurring performance. We successfully identified a novel homozygous variant of the *COL2A1* gene in a Chinese patient with type II collagenopathy. The variant c.3662C > T was located in exon 51 and could result in a serine-to-phenylalanine substitution in the C-terminal region. Both parents of the proband were heterozygous for this variant. The allele frequency of the variant was absent from the gnomAD database (http://gnomad.broadinstitute.org/) and the 1000 Genomes Project (http://www.1000genomes.org). To the best of our knowledge, this is the first report of a variant resulting in serine replacement in *COL2A1*. Additionally, pathogenicity confirmed by in silico studies and the highly conserved protein and nucleotide sequences implied that this variant was disease-causing. Though the variant is classified as a variant of uncertain significance according to ACMG guidelines, a high correlation between this variant and the mild phenotype of type II collagenopathy was observed.

**Fig. 4:**
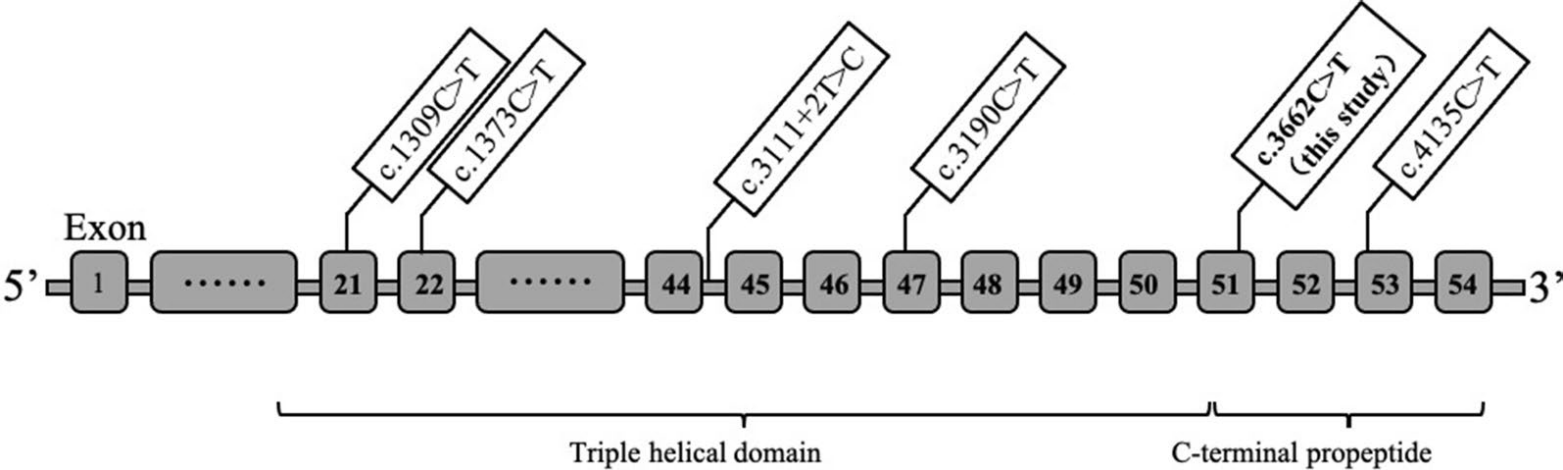
The schematic diagram of the distribution of 5 reported variants as well as c.3662C > T in the *COL2A1* gene

To date, 514 variants of the *COL2A1* gene have been reported in the human gene mutation database (http://www.hgmd.cf.ac.uk/ac/), including 241 missense variants, 95 splicing variants, 31 nonsense variants, 93 small deletions, 32 small insertions, and 22 other variants such as gross deletions/ insertions /duplications and complex rearrangements. As mentioned above, mutations in the *COL2A1* gene are mainly autosomal dominant although an autosomal recessive inheritance manner was reported in recent years. All reported *COL2A1* variants with autosomal recessive inheritance are shown in Fig. 4.

To demonstrate a genotype–phenotype correlation, we collected the clinical information of all patients with homozygous missense variants (Table 1) and analyzed the data by domains (Additional file 1: Table S3). We also compared these data with that of heterozygous variants.

**Table 1:** Clinical manifestations of patients with type II collagenopathies with homozygous mutations

Family	1	2	3	4		5	
Patient	1	2	3	4	5	6	7
Sex	Male	Male	Male	Male	Male	Female	Male
Age at onset	1.5 years	2 years	24 weeks (gestational age)	nd	nd	3 years	10 years
Age at diagnosis (genetic confirmation)	5 years	nd	4 years	24 years	22 years	7 years	10 years
Consanguinity	–	+	+	+	+	+	+
Ethnicity	Chinese	Indian	nd	Indian	Indian	Indian	Indian
Birth height (cm)/weight (kg)	51 (+ 0.33SD)/4 (+ 1.6SD)	49 (− 0.8SD)/3.1 (− 0.9SD)	38.5 (− 4.5SD)/2.7 (− 1.5SD)	nd	nd	nd	nd
Height (cm)/weight (kg) at last evaluative	105.8 (− 1.29)/24 (+ 1.87)	117.5 (− 4.8SD)/nd	68 (− 4SD)/9.5 (− 4.5)	97 (− 11)/nd	104 (− 10)/nd	127 (− 2)/20 (− 2.8)	111 (− 2.5)/17.5 (− 2)
Eyesight abnormality	–	+ (–6.5/–7.5D)	+ (–10D)	–	–	+ (+ 2D)	+ (+ 2.5D)
Hearing imapairment	–	+	–	–	–	–	–
Short limbs	–	+	+	+	+	–	–
Brachydactyly	+	+	nd	+	+	–	–
Motor delay	–	+	nd	+	nd	–	–
Short neck	+	+	+	–	–	–	–
Flat face	+	+	nd	–	–	+	+
Irregular vertebral endplate	+	+	–	+	+	+	+
Platyspondyly	+	+	+	+	+	+	+
Lumbar lordosis	–	nd	nd	+	+	+	+
Kyphosis	–	+	–	–	–	–	+
Scoliosis	–	+	–	–	–	+	+
Epiphyseal dysplasia	+	+	+	+	+	+	+
Metaphyseal enlargement	+	+	+	+	+	+	+
Joint pain/stiffness/laxity	+	nd	nd	+	+	+	+
Waddling gait	–	+	nd	–	–	+	+
Others		Pyloric stenosis, inguinal hernia, insulin resistance, Barrel shaped thorax	Pierre Robin sequence features (cleft palate, bifid uvula, retrognathia, glossoptosis)		Bilateral knee joint dislocation		
Identified variant	c.3662C > T (p.Ser1221Phe)	c.1309C > T p. (Arg437Trp)	c.1373C > T p. (Pro458Leu)	c.4135C > T p. (Arg1379Cys)	c.4135C > T p. (Arg1379Cys)	c.3190C > T p. (Arg1133Cys)	c.3190C > T p. (Arg1133Cys)
Exon	51	21	22	53	53	47	47
Domain	C‐terminal	Triple helical domain	Triple helical domain	C‐terminal	C‐terminal	Triple helical domain	Triple helical domain
Reference	This study	Tham, E., et al	Barat-Houari, M., et al	Girisha, K. M. et al			

nd, no data

Vertebral abnormality, such as irregular vertebral endplates and platyspondyly could be observed in almost all patients with homozygous missense variants. Platyspondyly is also a common feature in different phenotypes of type II collagenopathy inherited in a heterozygous manner [2]. Eyesight abnormality, kyphosis, scoliosis, and waddling gait only occur in patients with mutations in the triple-helical domain. However, in cases with heterozygous mutations, these symptoms are not specifically associated with mutations in the triple-helical domain. Additionally, glycine substitution was highly relevant to SEDC [11]. Glycine substitution usually leads to an abnormal conformation or destabilization of the triple helix, thereby acting in a dominant‐negative way [2]. No glycine substitution has been reported in patients with homozygous variants to date.

Variants in the C-terminal region could also affect collagen formation. Patients with mutations in the C-terminal region present with milder phenotypes with some characteristic symptoms, probably because the variant has limited influence on the mature type II collagen [11]. Brachydactyly, especially of the middle and distal phalanges, seems to be the most common clinical feature of variants in the C‐terminal region [3, 12], which was also observed in the present case. Further, variants in the C-terminal propeptide are also associated with platyspondylic skeletal dysplasia Torrance type(MIM#151210) and with Spondyloperipheral dysplasia (SPPD, MIM#271700) [13]. Based on the present case, serine substitution in the C-terminal region seems to have no relation with severe phenotypes; however, there is not enough data reported on serine substitution at this time to make a definitive conclusion.

Our data collection and analysis may provide more insights into the phenotype of type II collagenopathy, especially for patients with homozygous missense variants. Tham et al. [5] reported the first patient with a homozygous variant and assumed the relationship between the bi-allelic variant of *COL2A1* and SEDC. This was supported by Barat‐Houari et al. [4], who described a more severe patient with SEDC with homozygosity. However, Girisha et al. [3] then reported four patients with bi-allelic variants in *COL2A1* which rarely caused SEDC. In type II collagenopathies dominantly inherited, more than 100 *COL2A1* variants have been reported in patients diagnosed with SEDC, and most of the variants are located in the triple-helical domain (74% Gly replacements and 10% Arg-to-Cys substitutions) [14]. Our patient had mild abnormalities of the vertebrae with no hearing or ocular involvement, which shows that the case had a limited correlation with SEDC. Similar to autosomal dominant cases, different domains and amino acid substitutions should be considered. SEDC is not a homozygous variant-specific phenotype.

We also noticed that the patient’s height was within the normal range, whereas all of the previously reported cases had short stature at the last evaluation. Tham et al. [5] described detailed height information of a patient from birth to 11 years of age; height at birth was variable. Short stature in recessively inherited patients seems to become more severe as the patient gets older, indicating that height influence on the patient is probably cumulative. However, in patients with heterozygous mutations in the C-terminal propeptide, height clustered around the average level [13]. There is no specific relationship between height and domains in patients with homozygous variants. The proband’s height should be monitored closely.

It should be noted that all patients with homozygous variants were from consanguineous families in West Asia, except for our patient. Thus, the carrying rate of *COL2A1* in Chinese may be underestimated.

In conclusion, this study reported the first Chinese patient with type II collagenopathy with autosomal recessive inheritance, thereby enriching the spectrum of genotypes. The patient presented with a mild phenotype. With the rapid development and application of sequencing technologies, we believe that more variants relating to milder phenotypes will be identified. However, direct functional evidence is lacking to prove the pathogenicity of all variants inherited in an autosomal recessive manner. The precise genotype–phenotype correlation and specific mechanisms remain unknown and require further study.

## Supplementary Information

**Additional file 1:Table S1.** Variants filtering processes . **Table S2.** Significant variants found in the patient. **Table S3.** Clinical manifestations of patients with type II collagenopathies with homozygous mutations in different domains. **Figure S1.** Radiographs of the proband’s father. A, B. The cervical spine. C, D. The thoracic spine. F. The right pelvis. E, G. The lumbar spine. **Figure S2.** Radiographs of the proband’s mother. A, B. The Tervical spine. C, D. The thoracic spine. E, F. The right knee. G, H. The lumbar spine. **Figure S3.** Radiographs of the proband’s brother. A, B. The cervical spine. C, D. The thoracic and lumbar spine. E, F. The right knee.

## Data Availability

The variant has been submitted to the NCBI ClinVar database whose accession number is SCV001755682. The raw sequence datasets generated during the current study are not publicly available because it is possible that individual privacy could be compromised but they are available from the corresponding author on reasonable request.

## Abbreviations

STL1: Stickler syndrome type I
SPPD: Spondyloperipheral dysplasia
SEDC: Spondyloepiphyseal dysplasia congenital
WES: Whole-exome sequencing
ACMG: American College of Medical Genetics and Genomics
