# Supplementary material for: A novel homozygous variant of COL2A1 in a Chinese male with type II collagenopathy: a case report
Source: BMC Med Genomics. 2021 Aug 11;14:201. doi: 10.1186/s12920-021-01048-0 (PMC8359039; doi:10.1186/s12920-021-01048-0)
Supplement: Supplementary file 1 — Additional file 1:Table S1. Variants filtering processes . Table S2. Significant variants found in the patient. Table S3. Clinical manifestations of patients with type II collagenopathies with homozygous mutations in different domains. Figure S1. Radiographs of the proband’s father. A, B. The cervical spine. C, D. The thoracic spine. F. The right pelvis. E, G. The lumbar spine. Figure S2. Radiographs of the proband’s mother. A, B. The Tervical spine. C, D. The thoracic spine. E, F. The right knee. G, H. The lumbar spine. Figure S3. Radiographs of the proband’s brother. A, B. The cervical spine. C, D. The thoracic and lumbar spine. E, F. The right knee. [file 12920_2021_1048_MOESM1_ESM.docx]

Supplementary Table S1. Variants filtering processes

| Genome | Homo_sapiens_97 |
| --- | --- |
| Date | 2019/12/13 12:29 |
| Average read depth | 162.91 |
| Number of variants (before filter) | 26,670 |
| Exclude UTR and deep intronic variants | 17,748 |
| Exclude synonymous variants | 10,475 |
| Exclude variants with high population frequency (gnomad_genome_AF>0.001) | 3202 |
| Exclude variants located in non-OMIM genes | 1,076 |
| Exclude variants by HPO terms related genes (HP:0000924,Abnormality of the skeletal system) | 214 |

Supplementary Table S2. Significant variants found in the patient

| Gene name | position | cDAN | protein | Genotype | ENSG num | ENST_Number | Exon_CDS | Depth | Revel_Score | ClinPred_Score | ada_score | mmsplice_delta_logit_psi | dpsi_zscore | gnomad_exon_AF | gnomad_genome_AF | |
| --- | --- | --- | --- | --- | --- | --- | --- | --- | --- | --- | --- | --- | --- | --- | --- | --- |
| COL2A1 | chr12-48369324-g-a | c.3662C>T | p.Ser1221Phe | hom | ENSG00000139219 | exon51 | ENST00000380518.3\|missense_variant\|MODERATE\|protein_coding\|51/54\|c.3662C>T\|p.Ser1221Phe\|3662/4464\|1221/1487 | 0/189(1.0) | | - | 0.635 | 0.934825 | - | - | - | - |
| FBN2 | chr5-127636602-c-t | c.6073G>A | p.Gly2025Ser | het | ENSG00000138829 | exon48 | ENST00000262464.4\|missense_variant\|MODERATE\|protein_coding\|48/65\|c.6073G>A\|p.Gly2025Ser\|6073/8739\|2025/2912 | 41/47(0.53) | 0.621 | 0.480189 | - | - | - | 0.0000756 | 6.37E-05 |  |
| GLI3 | chr7-42012196-t-a | c.1843A>T | p.Thr615Ser | het | ENSG00000106571 | exon13 | ENST00000395925.3\|missense_variant\|MODERATE\|protein_coding\|13/15\|c.1843A>T\|p.Thr615Ser\|1843/4743\|615/1580 | 86/87(0.5) | 0.349 | 0.306007 | - | - | - | 0.0000689 | 3.19E-05 |  |
| LRP4 | chr11-46920165-c-a | c.740G>T | p.Gly247Val | het | ENSG00000134569 | exon7 | ENST00000378623.1\|missense_variant\|MODERATE\|protein_coding\|7/38\|c.740G>T\|p.Gly247Val\|740/5718\|247/1905 | 270/194(0.42) | 0.555 | 0.580825 | - | - | - | - | - |  |
| LRP4 | chr11-46920165-c-a | c.740G>T | p.Gly247Val | het | ENSG00000134569 | exon7 | ENST00000378623.1\|missense_variant\|MODERATE\|protein_coding\|7/38\|c.740G>T\|p.Gly247Val\|740/5718\|247/1905 | 270/194(0.42) | 0.555 | 0.580825 | - | - | - | - | - |  |
| NEK9 | chr14-75573300-ag-a | c.1432delC | p.Leu478fs | het | ENSG00000119638 | exon12 | ENST00000238616.5\|frameshift_variant\|HIGH\|protein_coding\|12/22\|c.1432delC\|p.Leu478fs\|1432/2940\|478/979 | 78/81(0.51) | 0 | 0 | - | - | - | 0.00000398 | - |  |
| PIEZO2 | chr18-10718237-g-c | c.4876C>G | p.Arg1626Gly | het | ENSG00000154864 | exon34 | ENST00000503781.3\|missense_variant\|MODERATE\|protein_coding\|34/52\|c.4876C>G\|p.Arg1626Gly\|4876/8259\|1626/2752 | 91/75(0.45) | 0.132 | 0.038367 | - | - | - | - | 0.000127 |  |
| PIEZO2 | chr18-10857034-c-t | c.668G>A | p.Gly223Glu | het | ENSG00000154864 | exon6 | ENST00000503781.3\|missense_variant\|MODERATE\|protein_coding\|6/52\|c.668G>A\|p.Gly223Glu\|668/8259\|223/2752 | 23/34(0.6) | 0.759 | 0.133699 | - | - | - | - | 0.000255 |  |
| SERPINF1 | chr17-1680557-ccggg-c | c.1076_1079delGGGC | p.Arg359fs | het | ENSG00000132386 | exon8 | ENST00000254722.4\|frameshift_variant\|HIGH\|protein_coding\|8/8\|c.1076_1079delGGGC\|p.Arg359fs\|1076/1257\|359/418 | 134/110(0.45) | 0 | 0 | - | - | - | - | - |  |
| SERPINF1 | chr17-1680565-g-a | c.1082G>A | p.Gly361Asp | het | ENSG00000132386 | exon8 | ENST00000254722.4\|missense_variant\|MODERATE\|protein_coding\|8/8\|c.1082G>A\|p.Gly361Asp\|1082/1257\|361/418 | 132/107(0.45) | 0.365 | 0.372038 | - | - | - | - | - |  |

Supplementary Table S3. Clinical manifestations of patients with type II collagenopathies with homozygous mutations in different domains.

|  | Triple helical domain | C‐terminal |
| --- | --- | --- |
| Total patients | 4 | 3 |
| Eyesight abnormality | 4/4 | 0/3 |
| Hearing imapairment | 1/4 | 0/3 |
| Short neck | 2/4 | 1/3 |
| Flat face | 3/3 | 1/3 |
| Short limbs | 2/4 | 2/3 |
| Brachydactyly | 1/3 | 3/3 |
| Metaphyseal enlargement | 4/4 | 3/3 |
| Irregular vertebral endplates | 3/4 | 3/3 |
| Platyspondyly | 4/4 | 3/3 |
| Lumbar lordosis | 2/2 | 2/3 |
| Kyphosis | 2/4 | 0/3 |
| Scoliosis | 3/4 | 0/3 |
| Epiphyseal dysplasia | 4/4 | 3/3 |
| Motor delay | 1/3 | 1/2 |
| Joint pain/stiffness/laxity | 2/2 | 3/3 |
| Waddling gait | 3/3 | 0/3 |


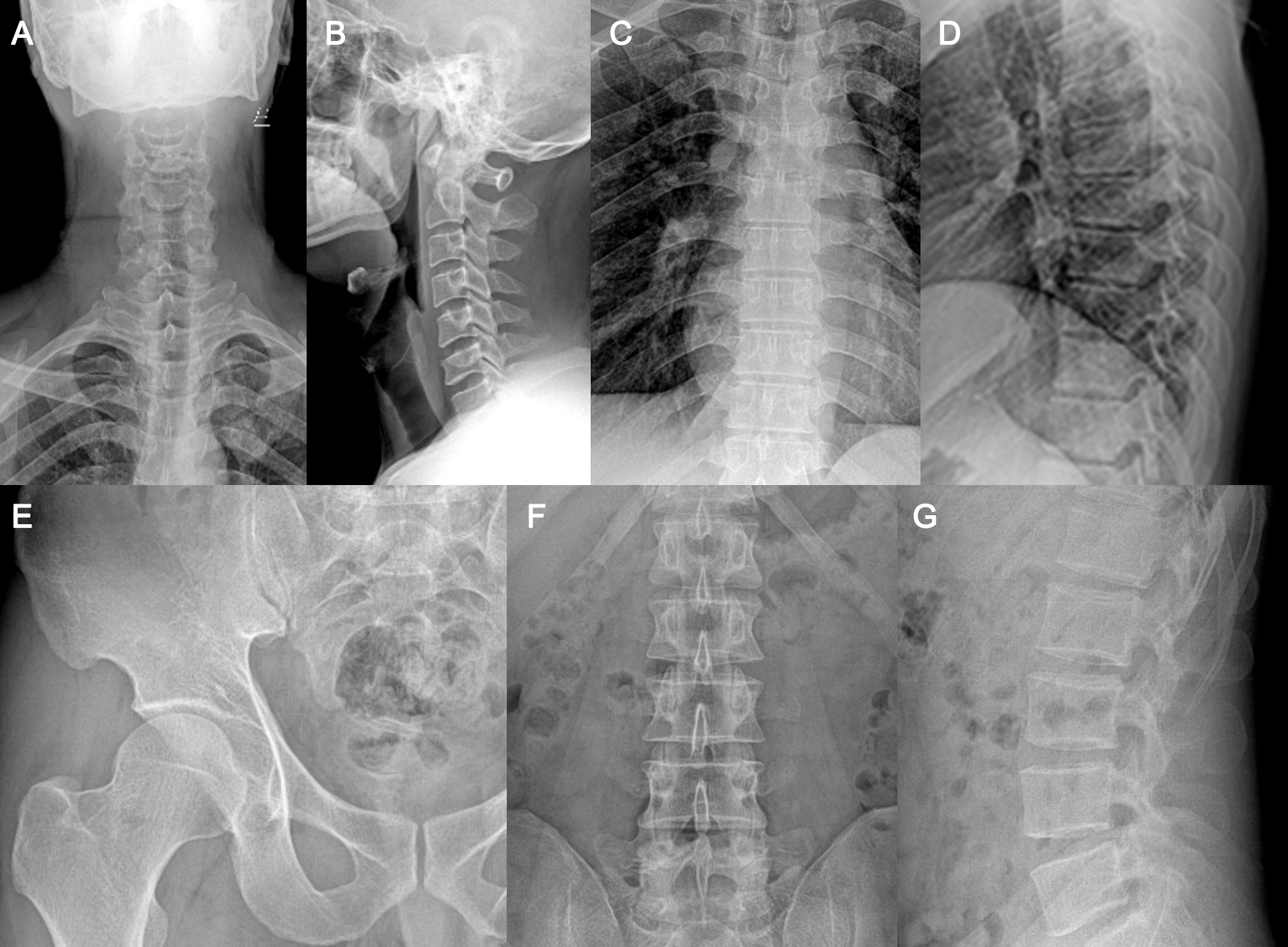


Supplementary Figure S1. Radiographs of the proband’s father. A, B. The cervical spine. C, D. The thoracic spine. F. The right pelvis. E, G. The lumbar spine.


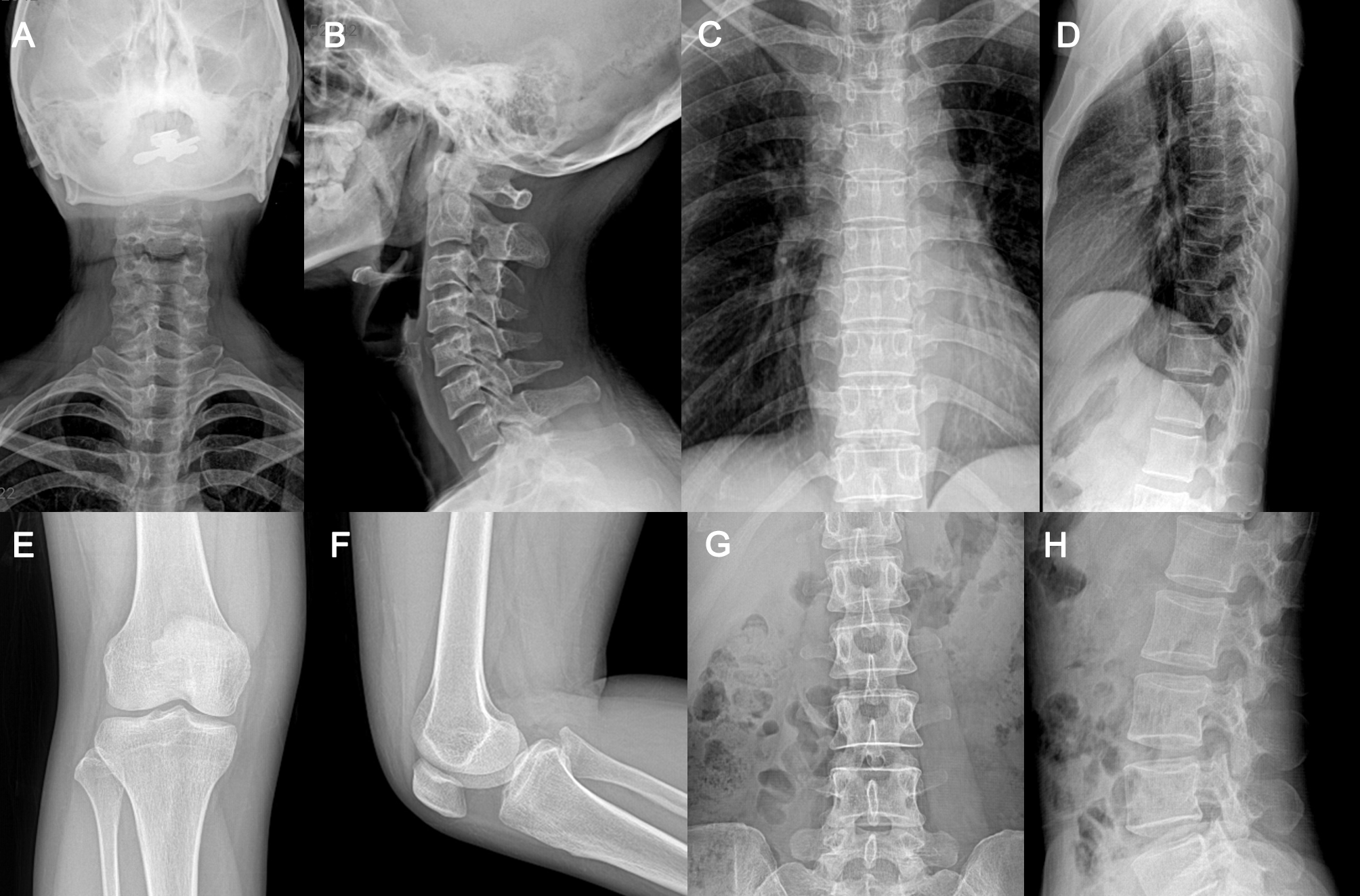


Supplementary Figure S2. Radiographs of the proband’s mother. A, B. The Tervical spine. C, D. The thoracic spine. E, F. The right knee. G, H. The lumbar spine.


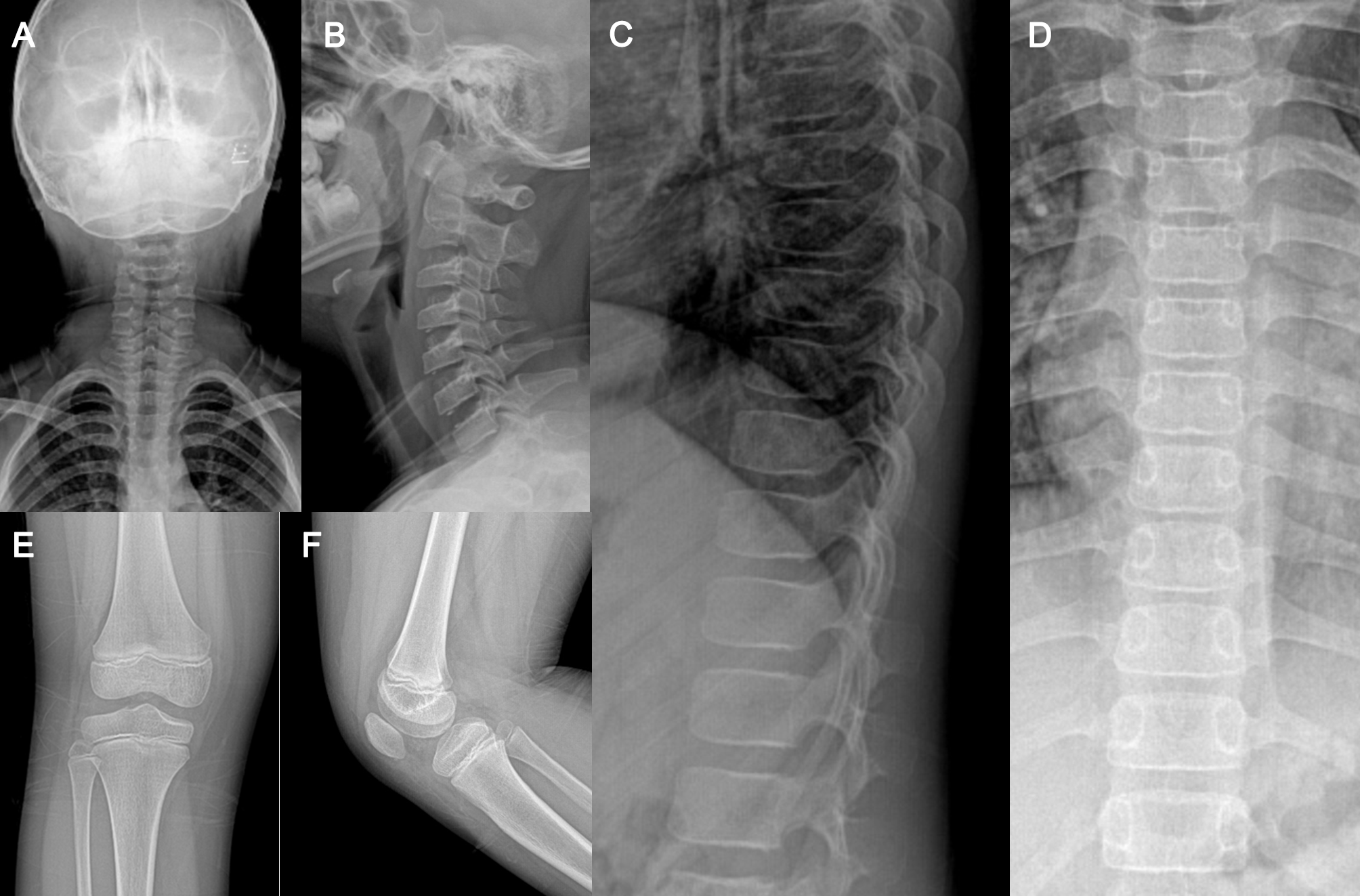


Supplementary Figure S3. Radiographs of the proband’s brother. A, B. The cervical spine. C, D. The thoracic and lumbar spine. E, F. The right knee.
